# Desire and Dread from the Nucleus Accumbens: Cortical Glutamate and Subcortical GABA Differentially Generate Motivation and Hedonic Impact in the Rat

**DOI:** 10.1371/journal.pone.0011223

**Published:** 2010-06-18

**Authors:** Alexis Faure, Jocelyn M. Richard, Kent C. Berridge

**Affiliations:** Department of Psychology, University of Michigan, Ann Arbor, Michigan, United States of America; Duke University, United States of America

## Abstract

**Background:**

GABAergic signals to the nucleus accumbens (NAc) shell arise from predominantly subcortical sources whereas glutamatergic signals arise mainly from cortical-related sources. Here we contrasted GABAergic and glutamatergic generation of hedonics versus motivation processes, as a proxy for comparing subcortical and cortical controls of emotion. Local disruptions of either signals in medial shell of NAc generate intense motivated behaviors corresponding to desire and/or dread, along a rostrocaudal gradient. GABA or glutamate disruptions in rostral shell generate appetitive motivation whereas disruptions in caudal shell elicit fearful motivation. However, GABA and glutamate signals in NAc differ in important ways, despite the similarity of their rostrocaudal motivation gradients.

**Methodology/Principal Findings:**

Microinjections of a GABA_A_ agonist (muscimol), or of a glutamate AMPA antagonist (DNQX) in medial shell of rats were assessed for generation of hedonic “liking” or “disliking” by measuring orofacial affective reactions to sucrose-quinine taste. Motivation generation was independently assessed measuring effects on eating versus natural defensive behaviors. For GABAergic microinjections, we found that the desire-dread motivation gradient was mirrored by an equivalent hedonic gradient that amplified affective taste “liking” (at rostral sites) versus “disliking” (at caudal sites). However, manipulation of glutamatergic signals completely failed to alter pleasure-displeasure reactions to sensory hedonic impact, despite producing a strong rostrocaudal gradient of motivation.

**Conclusions/Significance:**

We conclude that the nucleus accumbens contains two functional affective keyboards for amino-acid signals: a motivation-generating keyboard and a hedonic-generating keyboard. Corticolimbic glutamate signals and subcortical GABA signals equivalently engage the motivation keyboard to generate desire and-or dread. Only subcortical GABA signals additionally engage the hedonic keyboard to amplify affective “liking” and “disliking” reactions. We thus suggest that top-down cortical glutamate signals powerfully regulate motivation components, but are relatively unable to penetrate core hedonic components of emotion. That may carry implications of limits to therapeutic regulation of pathological emotions.

## Introduction

Local activation of GABA_A_ receptors or localized blockade of glutamate AMPA receptors in the medial shell of NAc each generate intense levels of motivated appetitive or fearful behaviors in an anatomically organized pattern of valence. Desire versus dread is generated by both GABAergic and glutamatergic microinjections along a rostrocaudal gradient in medial shell, in a manner analogous to a limbic ‘affective keyboard’ [1], [2], [3], [4], [5], [6]. Just as a keyboard generates many notes, neurochemical manipulations at different rostrocaudal points in medial shell generate many graded combinations of appetitive and/or defensive behaviors [3], [4], [5].

For example, in the rostral 25% of medial shell, microinjections of the GABA_A_ agonist muscimol or the glutamate AMPA-kainate antagonist DNQX each generate high levels of pure appetitive behaviors such as eating or drinking [1], [7], [8], [9], [10]. By contrast, near the rostrocaudal midpoint of shell muscimol or DNQX microinjections generate bivalent mixtures of both appetitive and fearful reactions. The fearful reactions include species-specific defensive behaviors such as distress vocalizations, escape attempts, conditioned place avoidance and defensive treading [2], [3], [4], [5], [6]. Defensive treading in particular occurs in the wild as an instinctive anti-predator behavior used to kick sand at snakes or other natural threats, and in the laboratory to bury or build protective mounds against small localized shock prods or other noxious objects [5], [6], [11], [12]. In the caudal 25% of medial shell, high levels of these fearful behaviors are elicited relatively purely by microinjections of the same drugs, whereas appetitive behaviors become suppressed [5], [6].

**Figure 1:**
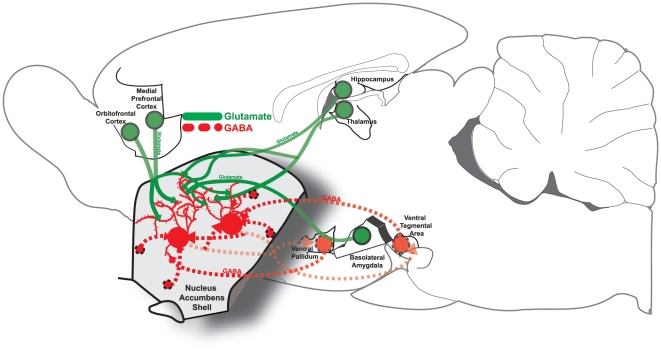
Glutamatergic and GABAergic NAc Circuits. Green; glutamatergic inputs from medial prefrontal cortex, orbitofrontal cortex, hippocampus, thalamus, and basolateral amygdala are shown entering the NAc, where they synapse on distal dendrites of medium spiny neurons in NAc. Red; GABAergic inputs from ventral pallidum and ventral tegmental area, as well as GABAergic interneurons and axon collaterals from other medium spiny neurons are shown synapsing onto proximal dendrites and soma of medium spiny neurons within NAc.

Therefore, similar appetitive-fearful behaviors are generated by glutamatergic and GABAergic microinjections at appropriate points along this rostrocaudal gradient in medial shell. But glutamate and GABA signals in NAc differ in important respects. Glutamate blockade in medial shell primarily blocks the impact of excitatory glutamate release by cortical-related projections from regions of neocortex (e.g., prefrontal cortex), and from cortical-type forebrain structures (e.g., basolateral amygdala and hippocampus), and corticolimbic relay nuclei (e.g., thalamus paraventricular nucleus) [1], [13], [14], [15], [16], [17] (Fig. 1). GABA signals arise primarily from subcortical circuits, and muscimol more directly modulates intrinsic spiny neurons of medial shell, mimicking inputs from collateral axons from other spiny NAc neurons and from ventral pallidum projections and projections from the ventral tegmental area, and related subcortical sources [1], [18], [19], [20], [21], [22] (Fig. 1). These differences suggest that GABA signals to NAc function essentially as bottom-up signals whereas glutamate signals function more heavily as top-down glutamate signals. These differences may carry functional implications for the generation of desire and dread emotions, despite their outward similarities in motivated behavior of eating versus defensive treading, distress calls, etc.

To probe this issue, here we contrasted GABAergic versus glutamatergic manipulations of medial shell for generation of 1) hedonic ‘liking’ shifts, measured in affective reactions to sucrose-quinine tastes, and 2) motivation gradients of appetitive and fearful behaviors as described above. We report that despite generating comparable levels of fear and feeding behaviors, only GABA manipulation in medial shell simultaneously shifts the hedonic impact of a sensory pleasure or displeasure. By contrast, glutamate disruption leaves hedonic impact unchanged. These results suggest that corticolimbic glutamate inputs to medial shell can produce strong motivations but cannot penetrate as effectively into the hedonic pleasure or displeasure components of emotions generated by subcortical circuitry.

## Results

### Synopsis

**Figure 2:**
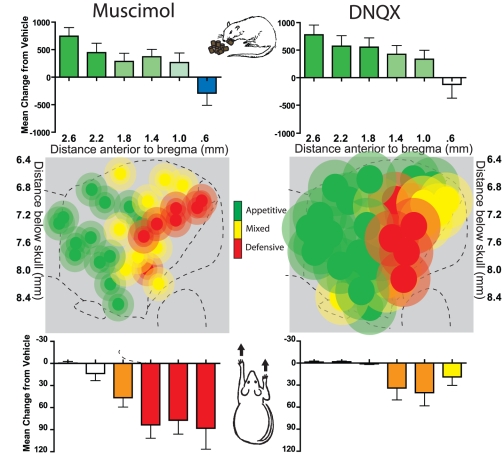
Summary maps of appetitive versus defensive motivation produced by GABA-A agonism and AMPA antagonism. Fos plume maps of appetitive eating versus defensive treading behavior generated by muscimol GABA stimulation (left) or DNQX AMPA blockade (right). Sites were designated as producing primarily appetitive (green symbols), defensive (red symbols) or mixed (yellow symbols) motivated behavior following muscimol and DNQX microinjections. Purely appetitive behavior (criteria for including a site was a >400 sec increase in feeding behavior) was primarily stimulated in rostral shell by both DNQX and muscimol, whereas defensive behavior (criteria for including a site was a >15 sec increase in treading behavior over vehicle levels) was primarily stimulated in caudal shell by both DNQX and muscimol. Histograms bars show mean change from vehicle (error bars  =  SEM) for both feeding (top) and defensive treading (bottom) at all rostrocaudal levels.

The effect of medial shell modulation of either GABAergic and glutamatergic transmission was assessed, for distinct group of animals, either on hedonic ‘liking’ affective response to sucrose-quinine taste using the taste reactivity test or on generation of appetitive or aversive motivated behavior expressed as spontaneous emission of naturalistic eating versus defensive treading behaviors. In this design both GABAergic and glutamatergic modulation was conducted in the same animals, allowing us to compare specific effects of each drug on behavioral tests in the same rat. Both muscimol, the GABA agonist, and DNQX, the glutamate AMPA antagonist, produced similar rostrocaudal gradients of appetitive and defensive behaviors that conformed to an ‘affective keyboard’ pattern (Fig. 2).

**Figure 3:**
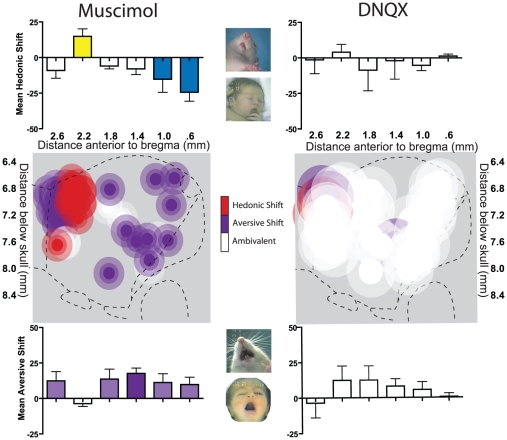
Summary maps of hedonic and aversive shifts produced by GABA-A agonism and AMPA antagonism. Fos plume maps of sites where muscimol GABA stimulation (left) or DNQX AMPA blockade (right) produced hedonic (red symbols) or aversive (purple symbols) in orofacial reactions to a sweet/bitter sucrose-quinine mixture at 15 minutes post-microinjection. Criteria for including a site as hedonic was an increase >10 in ‘liking’ reactions and a decrease or no change in ‘disliking’ reactions. Criteria for including a site as aversive was an increase >10 in ‘disliking’ reactions and/or a decrease >10 in ‘liking’ reactions. Hedonic enhancement was produced by muscimol only a moderately rostral area just below the genu of the corpus callosum. Aversive enhancement was produced by muscimol throughout rostral shell, and in an area rostral to the hedonic enhancement zone, possibly extending into rostral pole of NAc. DNQX produced mostly ambivalent effects (white; no change, or simultaneously enhanced or suppressed ‘liking’ and ‘disliking’ reactions). Histograms bars show mean change from vehicle (error bars  =  SEM) for both hedonic ‘liking’ reactions (top) and aversive ‘disliking’ reactions (bottom) at all rostrocaudal levels.

However, only stimulation of GABA receptors with muscimol microinjection generated corresponding shifts in affective reactions to the hedonic impact of sweet or bitter tastes, whereas glutamate disruptions had no effect on hedonic impact (Fig. 3). For example, bittersweet tastes became more positively ‘liked’ (e.g., elicited more lip licking and similar hedonic orofacial reactions) after rostral shell microinjections (34.3+/−5.88 SEM hedonic reactions on muscimol versus 19.5+/−6.45 SEM on vehicle, F(1,5) = 7.888, p = .038), but became negatively ‘disliked’ (e.g., elicits gapes) after caudal microinjections (2.00+/−.632 SEM hedonic reactions on muscimol versus 20.8+/0 5.68 SEM on vehicle, F(1,9) = 10.970, p = .009; 25.3+/−2.39 SEM aversive reactions on muscimol versus 12.9+/−2.75 SEM on vehicle, F(1,9) = 12.880, p = .006). Glutamate AMPA blockade with DNQX microinjections completely failed to alter hedonic ‘liking’ or aversive ‘disliking’ reactions to sensory pleasure or displeasure, despite generating spontaneous motivated fearful or feeding behaviors at levels similar to GABAergic microinjections.

### Fos plume analysis of local drug impact

**Figure 4:**
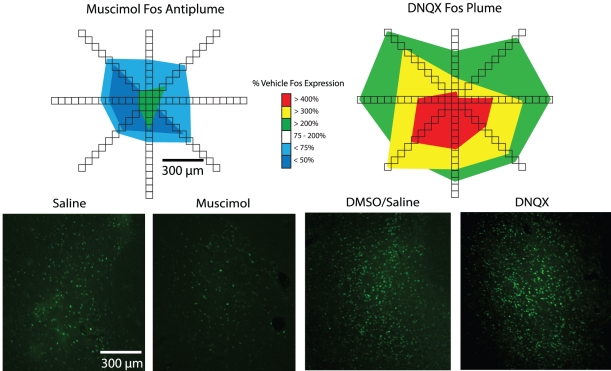
Fos plume examples for muscimol and DNQX. Colored plume maps for both muscimol (left) and DNQX (right) show local elevations or suppressions of Fos caused relative to vehicle (top). Muscimol produces an inhibitory “anti-plume” or an area of suppressed Fos expression (to 75% and 50% of vehicle levels), surrounding a small excitatory center of 200% vehicle levels. DNQX produces a larger, excitatory plume with areas of 200–400% vehicle level Fos expression. Examples of Fos expression produced by both vehicles (saline and the 50% saline/50% DMSO mixture) as well as muscimol and DNQX are shown on the bottom.

In order to map where microinjections were likely to have directly impacted local tissue, and to assign anatomical responsibility for behavioral effects, we used a Fos plume tool applied to separate rats to measure local plume-shaped regions of neuronal modulation caused by microinjections of DNQX, muscimol or each of the vehicles (total n = 33) [23], [24], [25] (Fig. 4). Local plumes of Fos expression provide a relatively direct measure of the spread of drug impact on local brain tissue, in the form of immediate early gene transcription and translation within nearby neurons.

We used a split-and-recombine design to compare behavioral and neurobiological Fos plume effects of GABA versus glutamate drug microinjections [23], [24], [25]. This procedure avoids under-estimation of drug spread that could result if plumes were assessed after a series of behavioral tests, due to progressive gliosis induced by microinjection repetition. Rats were assigned to either behavioral or Fos analysis groups at the time of surgery.

Each DNQX plume contained a ∼<0.08 mm^3^ volume center of quadrupled Fos expression levels (compared to vehicle levels; DNQX center radius  = 0.267+/−0.042 mm SEM). The plume center was surrounded by a middle band of tripled Fos expression (radius  = 0.528+/−0.068 mm SEM), which was surrounded by an outer rim of doubled Fos levels (radius  = 0.978 mm).

Muscimol (75 ng) microinjections produced small, inhibitory “antiplumes” [23] of 0.3 mm radius, where local Fos expression was suppressed below vehicle levels, surrounding an even smaller excitatory center, consistent with previous reports of muscimol-induced changes in Fos expression [26]. The very small excitatory center of doubled Fos expression (volume  = ∼0.004 mm^3^; radius  = 0.097+/−.016 mm SEM) was surrounded by the larger inhibitory antiplume of <½ Fos normal expression (compared to vehicle levels; volume ∼0.04 mm^3^; radius  = .209+/−0.032 mm SEM), and further surrounded by an outer weaker anti-plume of <75% vehicle Fos expression (volume  = ∼0.11 mm^3^; radius  = .304+/−.0468 mm SEM).

In comparison, DNQX produced slightly larger Fos plumes than muscimol (though reversed in polarity). The larger volume of the DNQX plume may perhaps be due to the fact that DNQX was dissolved in a more lipophilic vehicle (DMSO-saline mixture rather than pure saline), which may have produced greater diffusion.

### GABAergic muscimol evokes a gradient of “liking” versus “disliking” in medial shell

Muscimol microinjections shifted the positive hedonic impact of tastes in a keyboard-like gradient pattern, at rostral levels enhancing positive hedonic reactions but at more caudal levels suppressing the same positive hedonic reaction and instead amplifying aversive reactions to tastes (correlation of hedonic reaction change with rostrocaudal position  =  r (24) = 0.461, p<0.01).

Muscimol microinjection into a relatively far rostral level, about 0.5 mm thick and located at the level of the genu of the corpus callosum (+2.0 to +2.4 mm ahead of bregma) *enhanced* positive-valence hedonic reactions that are normally elicited by sucrose taste. Microinjection of muscimol at this rostral level in medial shell nearly doubled hedonic reactions to the sucrose-quinine mixture (muscimol  = 34.3+/−5.88 SEM versus vehicle  = 19.5+/−6.45 SEM tested at 15 min after microinjection; F(1.5) = 7.888, p = .038). The affective keyboard appeared to have an anterior end, so that hedonic reactions were no longer significantly enhanced if muscimol sites were moved further anterior, beyond this +2 to +2.4 rostral level (site X drug interaction, F(1,13) = 9.516, p = .009). That is, if microinjections were instead below the minor forceps of the corpus callosum, between +2.4 to 2.8 mm ahead of bregma, muscimol actually non-significantly decreased hedonic reactions to the taste mixture (average of 12.0+/−4.66 SEM versus 21.1+/−5.34 SEM under vehicle, F(1,7) = 2.835, p = .136). This far rostral zone anterior to +2.4 bregma extends into the rostral pole of NAc, and thus may be outside what is traditionally considered the medial shell. The rostral pole is further anterior than our previous fear and feeding gradient studies have typically mapped. These results suggest that the rostral pole is not part of the affective keyboard or functional rostrocaudal gradient for amplifying affective reactions.

**Figure 5:**
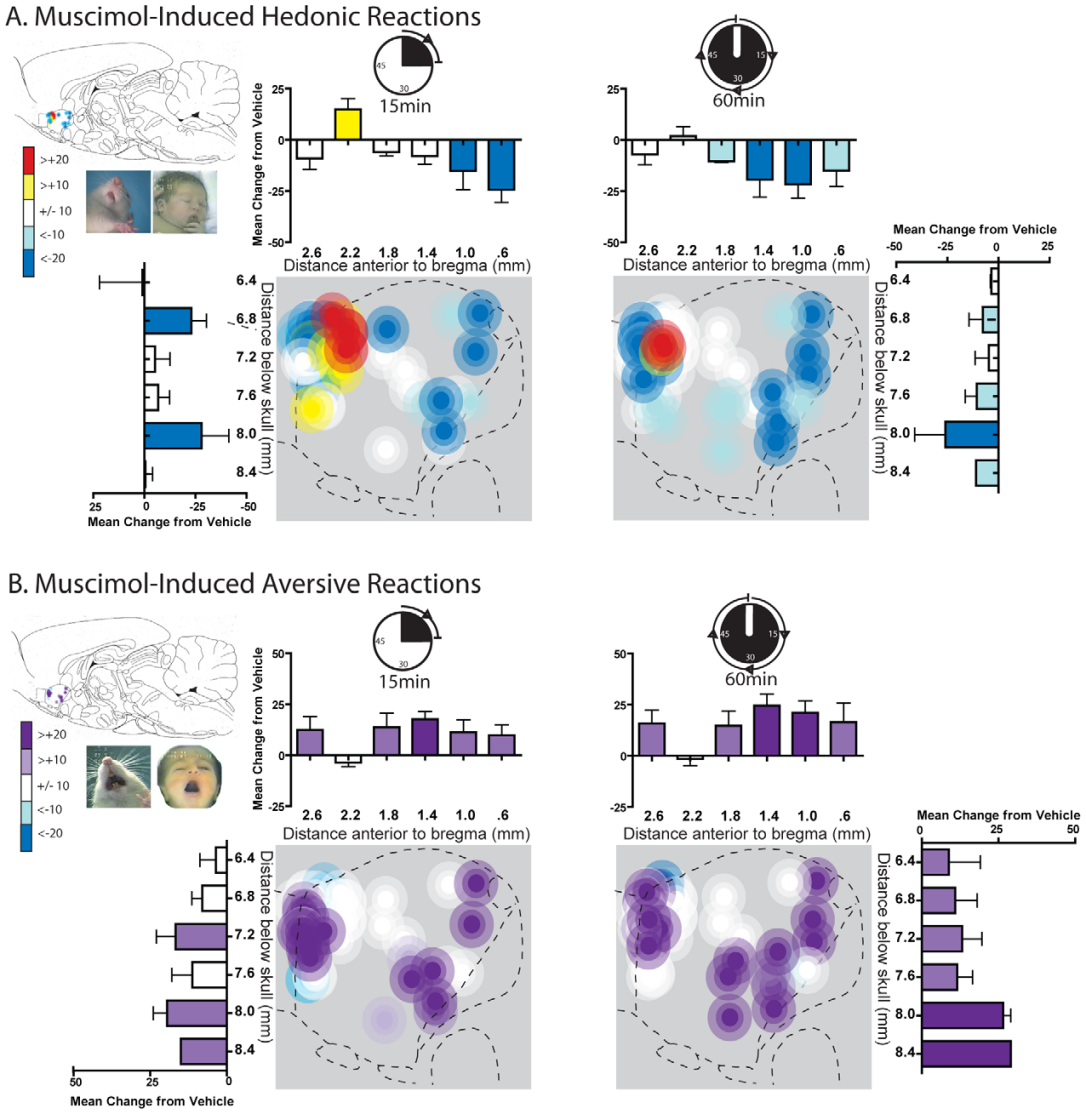
Muscimol induced changes in hedonic and aversive reactions. Fos plume maps of sites where muscimol GABA-A stimulation produced changes in hedonic ‘liking’ reactions (A) or aversive ‘disliking’ reactions (B) to sucrose-quinine taste at 15 (left) and 60 minutes (right) post-microinjection. Muscimol primarily enhanced ‘liking’ reactions in a small moderately rostral area, beneath the genu of the corpus callosum. Muscimol microinjection at sites outside of this area tended to reduce hedonic ‘liking’ reactions and enhanced aversive ‘disliking’ reactions, especially in caudal regions of NAc medial shell. Histograms bars show mean change from vehicle (error bars  =  SEM) for both each reaction pattern at rostrocaudal and dorsoventral levels throughout medial shell.

When sites were moved posteriorly into the caudal half of shell, muscimol microinjection suppressed or nearly abolished positive-valence hedonic reactions that are normally emitted to sucrose taste (e.g., tongue protrusions and paw licking) at both 15 min and 1 hr after microinjection (15 min: F(1,9) = 10.970, p = .009; 1 hr: F(1,10) = 11.962, p = .006; Fig. 3, 5, Fig S1). Supporting a keyboard-like pattern of affective modulation, muscimol produced progressively greater suppression of positive hedonic reactions elicited by oral infusions of sucrose-quinine solution as sites became progressively more caudal (correlation of hedonic suppression with caudal placement: r(25) = .461, p = .023).

Simultaneously, in caudal shell, muscimol microinjections dramatically increased the number of negative-valence aversive reactions (gapes, headshakes, forelimb flails), which are normally emitted to quinine taste (15 min: F(1,9) = 12.880, p = .006; 1 hr: F(1,10) = 11.165, p = .007, Fig. 3, 5, Fig S1). The number of aversive reactions progressively increased as sites became more caudal, and nearly doubled above vehicle levels at the most caudal sites (muscimol  = 25.3+/−2.39 SEM; vehicle  = 12.9+/−2.75 SEM). In summary, muscimol microinjection in medial shell generated a rostrocaudal gradient of rostral ‘liking’ and disliking ‘disliking’, corresponding to the concept of an affective keyboard.

### DNQX fails to clearly modulate “liking” or “disliking” reactions

**Figure 6:**
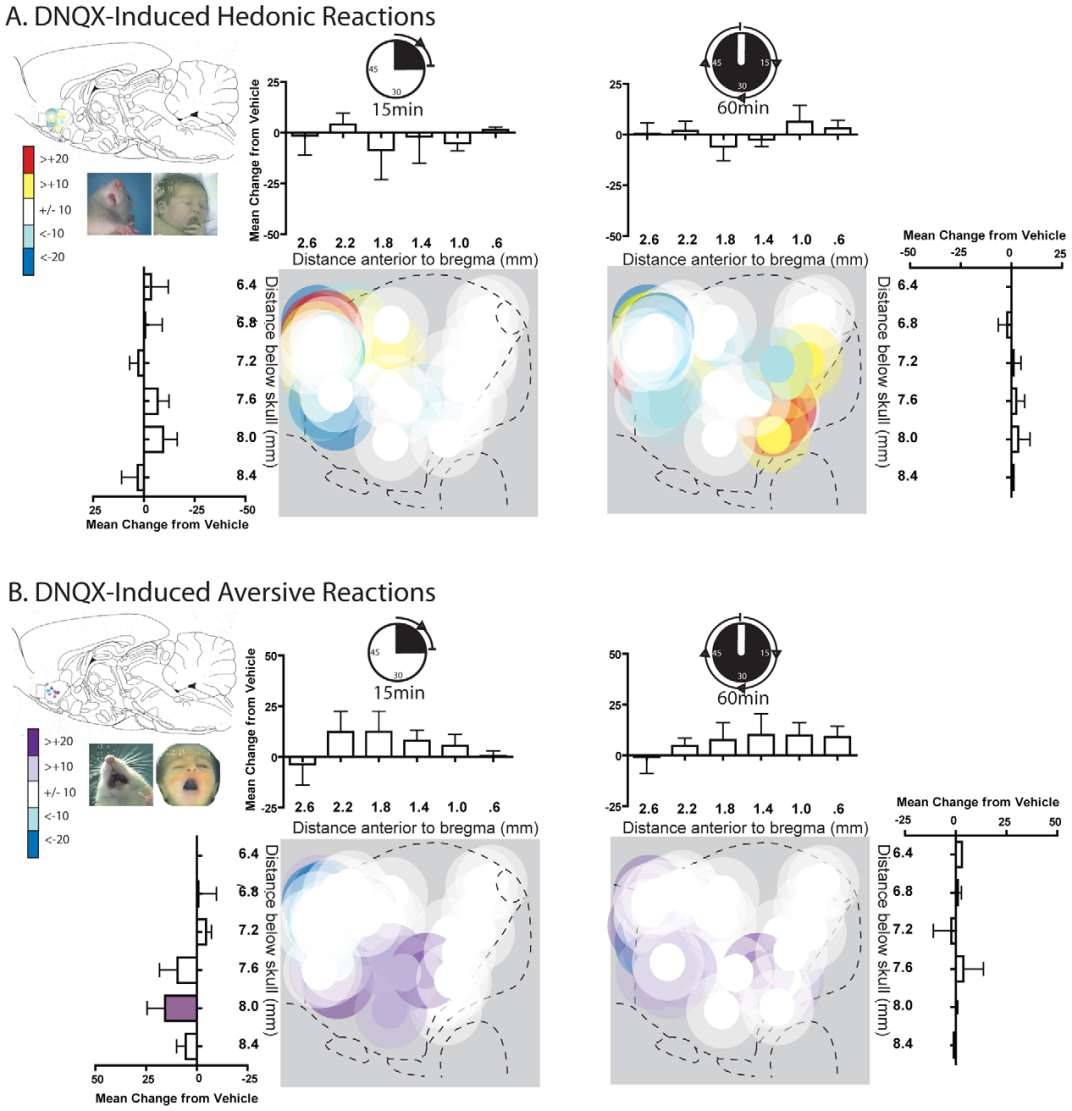
DNQX induced changes in hedonic and aversive reactions. Fos plume maps of sites where DNQX AMPA blockade produced changes in hedonic ‘liking’ reactions (A) or aversive ‘disliking’ reactions (B) to sucrose-quinine taste at 15 (left) and 60 minutes (right) post-microinjection. DNQX had little to no effect on both hedonic ‘liking’ reactions and aversive ‘disliking’ reactions. While some subjects did show changes in ‘liking’ or ‘disliking’ (as indicated by the sporadically colored symbols), no general pattern emerged. Histograms bars show mean change from vehicle (error bars  =  SEM) for both each reaction pattern at rostrocaudal and dorsoventral levels as marked along the medial shell.

By contrast, microinjections of DNQX in the shell completely failed to shift affective ‘disliking’ or ‘liking’ (Fig. 3, 6, Fig S1). Hedonic or aversive patterns of taste reactivity were never altered by DNQX microinjection at any rostrocaudal site (positive hedonic reactions, site X drug interaction, F(1,22) = .074, p = ns; aversive reactions, site X drug interaction, F(1,22) = .004, p = ns) or time after microinjection (positive hedonic reactions  = 15 min: F(1,23)  = .105, p  = ns; 1 hr: F(1,22) = .839, p = ns; negative aversive reactions  = 15 min: F(1,23)  = .934, p = ns; 1 hr =  F(1,22) = 1.560, p = ns). At most a few individual rats showed only noise-like fluctuations at random sites or tests after DNQX, without any discernable anatomical keyboard pattern or statistical significance.

### Direct contrast of DNQX to muscimol in the same rats for “liking” and “disliking”

As each rat was tested with both muscimol and DNQX at its particular anatomical site, it was possible to directly compare the effects on taste reactivity of the two neurochemical manipulations at the same rostrocaudal location in the same rat. Direct contrast of DNQX and muscimol effects confirmed that muscimol robustly altered hedonic reactions while DNQX did not (muscimol versus DNQX, site X drug interaction, 15 minutes, F(1,21) = 6.296, p = .020; 1 hr, F(1,21) = 6.957, p = .015). Similarly, muscimol amplified negative aversive reactions, but DNQX did not, at the same caudal sites (muscimol versus DNQX, caudal rats, F(1,9) = 8.741, p = .016).

### DNQX and muscimol induction of fear and feeding

**Figure 7:**
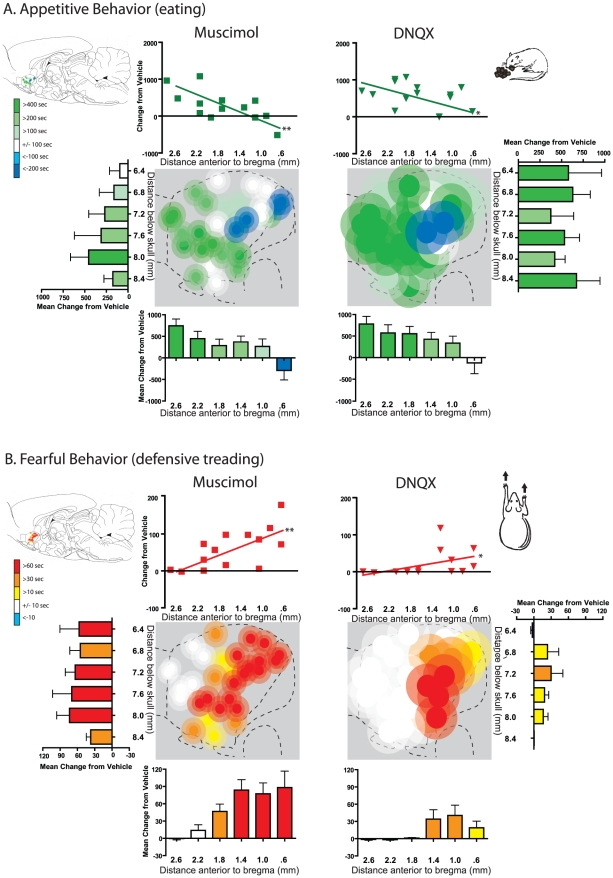
Muscimol and DNQX-induced feeding and defensive treading behavior. Fos plume maps of appetitive eating (A) and defensive treading (B) behavior generated by muscimol GABA stimulation (left) or DNQX AMPA blockade (right). Scatter plots above Fos plume maps indicate behavioral changes exhibited by individual subjects; lines indicate the slope of the correlation between behavioral change (from vehicle) and distance rostral to bregma in mm (*p<.05; **p<.01). Histograms bars below and to the sides of the maps show mean change from vehicle (error bars  =  SEM) for each behavior at rostrocaudal and dorsoventral levels as marked along the medial shell.

To confirm the presence of DNQX and muscimol-generated gradients of feeding and defensive behavior, one group of rats was tested only for the elicitation of spontaneous motivated behavior, along with a small subset of the rat that also went through taste reactivity testing. DNQX and muscimol generated similar patterns of feeding versus fear behaviors along the rostrocaudal gradient of motivation valence in medial shell. Rostral microinjections of either DNQX or muscimol at least quadrupled levels of appetitive eating behavior and food intake over vehicle control levels (feeding time, site X drug interaction, DNQX, F(1,14) = 8.589, p = .001, muscimol, F(1,12) = 8.159, p = .014; food intake, site X drug interaction, DNQX, F(1,14) = 6.996, p = .016, muscimol, F(1,12) = 7.129, p = .020, Fig. 2, 7). Progressively more rostral microinjections produced greater levels of feeding and intake (correlation with rostral distance from bregma for DNQX: r (16) = .528, p = .018; muscimol: r (14) = .747, p = .001). Microinjection of DNQX or muscimol into more caudal sites of medial shell did not increase feeding behaviors, and instead oppositely suppressed feeding behavior at the most caudal site for both drugs (DNQX, F(1,14) = 8.589, p = .001, muscimol, F(1,12) = 8.159, p = .014).

Conversely, caudal microinjections of either drug stimulated spontaneous generation of fearful behaviors such as robust defensive treading (site X drug interaction, DNQX, F(1,14) = 13.213, p = .003; muscimol, F(1,12) = 21.087, p = .001). As sites became progressively more caudal, the DNQX or muscimol microinjections produced increasingly higher levels of defensive treading behavior (correlation for DNQX: r (16) = −.462, p = .036; muscimol: r(14) = −.682, p = .004).

While both muscimol and DNQX microinjections produce similar patterns of feeding and fear behaviors, at rostral and caudal sites respectively, the intensity with which they did so differed slightly. There was a direct correlation between site placement along the rostrocaudal gradient with the intensity of motivated feeding versus fearful behaviors generated by microinjections, both for muscimol and DNQX (feeding time, r(14) = .603, p = .011; treading time, r(14) = .680, p = .004). Yet, DNQX produced slightly more appetitive eating behaviors at rostral sites (feeding time, site X drug interaction, F(1,12) = 7.084, p = .021), while muscimol produced more intense defensive treading behavior at caudal sites (treading time, site X drug interaction (F1,12) = 36.472, p<.001). In summary, both neurochemical manipulations produced similar rostrocaudal gradients for generating positive and negative motivated behaviors, and at similar overall intensities, but with slightly different valence biases (glutamate  =  positive motivation bias; GABA  =  negative bias).

We note that a limitation of the present results was that only one dose of DNQX was used to compare motivation and hedonic effects, and it would be valuable to confirm our conclusions with additional doses. However, the dose used was chosen to be maximally effective at generating appetitive and fearful motivations based on previous results [4], making it perhaps unlikely that other DNQX doses would modulate hedonic reactions any more potently than the dose used here.

## Discussion

Glutamate signals to NAc convey primarily top-down controls from cortical and cortex-related structures, such as prefrontal cortex, hippocampus, basolateral amygdala and thalamic nuclei embedded in cortico-limbic-thalamo-cortical loops. In contrast, GABA signals to the same medium spiny neurons in NAc convey primarily subcortical inputs from intrinsic local NAc neurons, and from other subcortical structures such as ventral pallidum. Here we showed that hyperpolarizing local disruptions of either corticolimbic glutamate or subcortical GABA inputs to NAc medial shell generate equivalent motivations expressed in behavior as appetitive desire and/or defensive dread mixtures, along the same rostrocaudal gradient in medial shell. But only GABA-related disruptions by muscimol additionally produced corresponding changes in the hedonic impact of an affect-laden gustatory stimulus. By contrast, glutamate-related disruptions by DNQX did not influence hedonic impact.

The valence of motivation (appetitive versus fearful) and of hedonic impact (pleasant versus unpleasant) generated by amino acid neurotransmitter disrupting microinjections was always determined by the rostrocaudal position of the microinjection site within the affective keyboard of NAc medial shell. In this keyboard pattern, each microinjection corresponded to a key, the size of which was the radius of the local Fos plume that surrounded the drug site. Each microinjection key was valence-tuned to a graded mixture of desire and/or dread corresponding to its rostrocaudal keyboard site in medial shell. Glutamate-related hyperpolarizations by DNQX actually produced a local plume of roughly three times larger radius (0.9 mm for outer radius of detectable Fos change) than GABA-related muscimol microinjections (0.304 mm radius), yet muscimol produced a broader array of functional effects.

DNQX microinjections and muscimol microinjections generated similar patterns of motivated fear or feeding behaviors. At rostral sites in medial shell, DNQX or muscimol each stimulated eating behavior and food intake to four times above vehicle control levels. Disruptions in medial shell completely failed to distort hedonic ‘liking’ or aversive ‘disliking’ reactions to sucrose-quinine taste, despite generating levels of motivated fearful or feeding behaviors intense as intense as muscimol. At caudal sites, DNQX or muscimol each generated fearful behaviors: chiefly defensive treading directed mostly at the front of the chamber and objects in the room beyond, at levels that often exceeded 10 times control vehicle levels. Eating behavior and food intake gradually declined and fearful behaviors gradually rose as sites moved progressively from rostral to caudal in medial shell.

However, only GABA disruptions generated a corresponding rostrocaudal gradient of shifted hedonic impact from sensory pleasure to displeasure. Muscimol microinjection at a rostral level enhanced positive ‘liking’ reactions to a bittersweet taste (e.g., lip or paw licking), whereas as sites moved caudally muscimol microinjections oppositely increased negative ‘disliking’ reactions (e.g., gapes) and suppressed positive ‘liking’ reactions. DNQX microinjections completely failed to alter ‘liking’ reactions to sweetness, and DNQX at sites in caudal shell completely failed to enhance negative ‘disliking’. Thus local glutamatergic AMPA blockade in medial shell powerfully induced motivated ‘wanting’ to eat, fearful anti-predator behaviors, or both, but never altered the hedonic impact of a sensory pleasure or displeasure.

The difference between effects on motivated desire-dread versus on hedonic ‘liking’-‘disliking’ reactions suggests that glutamate and GABA signals similarly can activate a motivation-generating keyboard in NAc to produce fear or feeding-related behaviors. But only GABA signals have additional access to a hedonic-generating keyboard corresponding to core affective reactions to sensory pleasure or displeasure.

### Neurobiological differences between GABAergic and glutamatergic microinjections

Muscimol microinjections stimulate GABA_A_ receptors and might be expected to produce hyperpolarization and reductions in the firing rate of local neurons containing GABA_A_ receptors. GABA_A_ stimulation by muscimol produces inhibitory hyperpolarizations by allowing Cl- to enter the cell [27], producing especially powerful inhibition of medium spiny neurons [28].

By comparison, DNQX microinjections block glutamatergic AMPA receptors, and might have been expected to similarly produce relative hyperpolarization of neurons containing glutamate receptors, by diminishing “up states,” suppressing EPSPs, and reducing the number of action potentials produced [29], [30], [31], [32], [33], [34], [35].

However, important differences also exist between GABAergic and glutamatergic hyperpolarizations. Muscimol may more potently hyperpolarize NAc neurons by acting on GABA_A_ receptors located on somata and proximal dendrites [36], [37], [38]. DNQX generation of desire and dread requires interaction with endogenous dopamine at the same site [3], [4], [5] and may act more distally on medium spiny dendrites, blocking ionotropic glutamate signals at distant spines, where AMPA receptors are more likely to be found [37], [39], [40], [41]. More distal placement of glutamate receptors on the head of neuronal spines, compared to GABA receptors, also might dilute the intensity of IPSP states at the soma and axon hillock induced by glutamatergic blockade, altering the degree of disinhibition passed on to output targets such as ventral pallidum, lateral hypothalamus or ventral tegmentum. The most potent hyperpolarizations may come via activation of fast-spiking interneurons which, in striatum, produce IPSCs in the postsynaptic neurons of 4 to 6 times the amplitude of those produced by medium spiny neurons [28]. One factor which may account for these intense differences in amplitude is synaptic location: parvalbumin-positive terminals, which likely arise from fast-spiking interneurons, are more likely to synapse on the soma, whereas medium spiny neurons are more likely to end on dendrites or spines [28], [42]. Generally, it seems the more proximal or intrinsic the input, the greater the inhibitory impact. All this implies that subcortical GABA inputs to NAc may achieve a greater potency of disinhibition of downstream targets to alter hedonic impact than glutamate inputs from predominantly cortical-related sources.

Finally, we note that muscimol microinjection produced “antiplumes” or areas of Fos suppression, whereas DNQX produced robust pure plumes of elevated Fos expression, consistent with our previous observations [2], [26]. These considerations suggest an additional qualitative difference between glutamatergic versus GABAergic hyperpolarizations, beyond a simple intensity difference, which may also contribute to differing modulation of hedonic reactions to sensory pleasure or displeasure.

As a caveat, it may be important to note that our conclusion that glutamate disruption fails to penetrate core ‘liking’ reactions to sensory pleasure is specific to ionotropic glutamate signals, and in particular to those requiring AMPA receptor activation. Blockade of AMPA receptors may be expected to disrupt ionotropic fast excitatory signals to medium spiny neurons in medial shell. This conclusion about fast-acting ionotropic receptors may need to be distinguished from metabotropic glutamate receptors that play a slower and broader modulatory role for neuronal function, and which could conceivably alter hedonic ‘liking’ more effectively than AMPA blockade, either directly by altering medium spiny neurons with metabotropic receptors or via presynaptic modulation of GABA release, which could in turn alter hedonic impact [43], [44]. Thus, we conclude that ionotropic AMPA glutamate disruptions in NAc shell do not modulate hedonic pleasure, but consider the hedonic role of metabotropic glutamate receptors to remain an open empirical question.

### Limits to top-down control?

An overall interpretation of our results may be that top-down corticolimbic inputs using glutamate signals from prefrontal cortex regions, such as infralimbic cortex (homologous in rats to ventral anterior cingulate cortex in humans), orbitofrontal or prelimbic cortex, or from hippocampus subiculum, basolateral amygdala, or paraventricular thalamus, all are limited in their ability to control hedonic emotional processes generated by NAc neurons, compared to bottom-up or subcortical inputs to the same NAc sites that primarily use GABA signals. Specifically, corticolimbic glutamate circuits appear to control the generation in medial shell of motivation components (incentive salience versus fearful salience), but not the generation of hedonic affective states (pleasure ‘liking’ versus displeasure ‘disliking’). This may restrict the capacity of top-down corticolimbic circuits to regulate subcortically generated emotion. Of course, another caveat is that our studies were conducted in rats, whereas primates and especially humans have larger prefrontal cortex and thus more dense glutamate projections to NAc. However, our results are still likely to apply to humans unless the quantitative species difference in top-down influence actually creates a qualitative expansion of control to include NAc-generated hedonics.

We conclude that the influence exerted by top-down controls over NAc may be limited to motivational states, and may leave core hedonic reactions to affective events relatively untouched. This feature might also conceivably set limits on the range of emotional processes that can be effectively adjusted by cognitive therapies that recruit top-down circuits [45], [46], [47], [48], [49], [50], [51], [52].

As caveat to our general distinction between cortical glutamate versus subcortical GABA sources, it is important to note one significant subcortical source of glutamate signals in NAc: co-release of glutamate by mesolimbic dopamine neurons [53]. Glutamate released by mesolimbic dopamine neurons can produce fast postsynaptic potentials in NAc that is blocked by DNQX, implicating ionotropic AMPA receptors [53]. It may be noteworthy that dopamine in NAc, like glutamate AMPA disruption, fails to enhance ‘liking’ reactions to sensory pleasure even when it elevates ‘wanting’, suggesting a functional similarity between ionotropic glutamate and dopamine actions in NAc [54], [55]. Also, endogenous dopamine is required for local AMPA glutamate blockade by DNQX to generate either motivated fear or feeding behaviors, again suggesting a synergistic interaction for dopamine-glutamate motivation effects in medial shell.

In contrast to glutamate disruption from any source, both motivation and hedonic impact are robustly generated together by GABAergic inhibition of neurons in medial shell, produced by inputs from neighboring medial spiny neurons and other intrinsic NAc neurons, or from GABAergic inputs from other subcortical structures [1], [56]. The ability of GABAergic inhibition to modulate hedonics is consistent with previous work on NAc generation of ‘liking’ and ‘disliking’, and also with related hedonic generation by the ventral pallidum, a major source of GABAergic input and output from NAc [23], [57], [58], [59].

### Conclusion

Both corticolimbic glutamate and subcortical GABA signals in medial shell can stimulate motivated behaviors reflecting appetitive and/or fearful motivations, organized rostrocaudally along an affective keyboard of desire versus dread in medial shell. At normal intensity levels, such amino acid neurotransmitter signals may act on valence-coded locations in rostral medial shell to help give healthy attractiveness to rewards and zest to life, whereas at excessively higher levels may contribute to compulsive drug addiction and related compulsive pursuits [56], [60]. Likewise, in caudal shell, GABA and glutamate signals may normally function to adaptively make threat-related stimuli frightening in appropriate situations, but at excessively higher levels contribute to levels of pathological paranoia in schizophrenia and related disorders [61], [62], [63], [64], [65], [66].

Yet despite the similarity of motivational effects of top-down and bottom-up signals using amino acid neurotransmitters, the core hedonic components of emotions generated in medial shell may differ qualitatively between them. Hedonic components of ‘liking’ reactions to pleasant sensations and ‘disliking’ reactions to unpleasant ones may be generated uniquely by subcortical inputs to the hedonic–generating keyboard for amino acid signals in medial shell, such as collaterals from neighboring intrinsic neurons, accumbens-pallidal loops, brainstem inputs and related projections [67], [68], [69], [70], [71], [72].

Our results are compatible with the hypothesis that the capacity of top-down corticolimbic circuits to regulate emotion generation has qualitative limits. We conclude that corticolimbic regulation of emotion might be more effective at modulating motivation components than at modulating hedonic reactions to the impact of emotional events.

## Materials and Methods

### Animals

Rats [n = 67 (behavioral testing, n = 36; Fos plume, n = 31), male, 280–320 g at surgery], were housed on a 12 hr light/dark reverse cycle (∼21°C) with ad libitum food (Purina Rat Chow) and water (tap water).

### Microinjection cannulae surgery

Rats were implanted bilaterally with stainless-steel guide cannulae at various rostrocaudal points in the medial shell of NAc. For each rat, the rostrocaudal point was arbitrarily assigned and bilaterally matched t to be symmetrical on left and right sides, but from rat to rat rostrocaudal assignments were staggered so that the group's placements as a whole filled the entire anteroposterior extent of medial shell. Rats were anesthetized with ketamine (80 mg/kg), xylazine (5 mg/kg), pre-treated with atropine (0.04 mg/kg), and positioned in a stereotaxic apparatus (David Kopf Instruments, Tujunga, CA). A slanted skull position was used to avoid penetrating the lateral ventricles, with the incisor bar set at 5.0 mm above interaural zero. Chronic bilateral microinjection guide cannulae (23 gauge; stainless steel) were positioned to end 2 mm above each target site in the medial shell [73]. Rostral shell placements were generally centered around the anteroposterior (AP) coordinates AP +3.4 (±1) mm ahead of bregma, caudal shell placements were centered around AP+2 (±1) mm ahead of bregma. In other dimensions, all mediolateral (ML) and dorsoventral (DV) coordinates were ML ±1 mm and DV-5.7 mm below the skull. Guide cannulae were anchored to the skull with four bone screws and acrylic cement, and stainless steel obturators were inserted into guide cannulae to prevent occlusion. A subset of rats (n = 26) designated for taste reactivity testing, also received implantation of oral cannulae for the infusion of taste solutions. Polyethylene tubing was inserted just lateral to the first maxillary molar and run subcutaneously along the zygomatic arch to the top of the skull, where it exited through an incision. There, the tubing was secured to 15 mm, 19 gauge stain-less steel cannulae with wiring and dental acrylic. Post-surgery, each rat received chloramphenicol sodium succinate (60 mg/kg) or prophylactic penicillin (aquacillin; 45, 000 U, i.m.) to prevent infection, and buprenorphine hydrochloride (0.3 mg/kg) for pain relief. Rats were allowed to recover for at least 7 d before behavioral testing.

### Drugs and Microinjections

Glutamate AMPA receptor blockade was achieved by microinjection of the AMPA/kainate glutamate receptor antagonist, DNQX (6,7-dinitroquinoxaline-2,3(1H,4H)-dione; Sigma, St. Louis, MO), dissolved in 50% DMSO/50% 0.15 M saline, at a dose of 450 ng/0.5 µl per side. This DNQX dose was the same used to produce rostrocaudal gradients of eating and defensive treading behaviors via microinjections into medial shell in recent studies [2], [3], [4]. GABA_A_ receptor activation was achieved by microinjections of muscimol (Sigma, St. Louis, MO) dissolved in 0.15M saline at 75 ng/0.5 µl per side, which was chosen according to previous results [5]. The third and fourth microinjection conditions were vehicle controls: the 50% DMSO/50% 0.15 M saline mixture used for DNQX solutions and the 100% 0.15M saline vehicle used for muscimol. The pH of all solutions was maintained between 7.0 and 7.4.

After 3 d of handling, rats were habituated to the test chambers for 3 consecutive days, and were given a vehicle microinjection (saline) on the last day of habituation. Each rat was subsequently tested with all 4 microinjection conditions in counterbalanced order, in test sessions spaced 48 hours apart. Microinjection cannulae (29 gauge) which extended 2.0 mm beyond the ventral tip of the guide, were attached to a syringe pump via PE-20 tubing and inserted into the guide cannulae. Rats were gently hand-held while they were bilaterally infused with a microinjection volume of 0.5 µl at a rate of 0.3 µl/min. After infusion, the injectors remained in place for an additional 60 sec to allow for drug diffusion before their withdrawal and replacement of the obturators. The rat was placed immediately into a behavioral testing chamber.

### Behavioral Taste Reactivity Tests

For taste reactivity tests, immediately following drug microinjection, a polyethylene delivery tube was connected to the rat's oral cannulae. Rats were placed into the test chamber, which had a transparent floor, under which an angled mirror reflected an image of the rat's ventral face and mouth into a digital video camera. At 15 min and 60 min post-microinjection, a solution containing a mixture of sucrose and quinine (0.1 M sucrose and 1.66×10^−4^ M quinine) was infused in 1 ml volume over a 1 min period via syringe pump connected to the delivery tube. The sucrose-quinine mixture was used in order to elicit both positive hedonic reactions (‘liking’) and negative aversive reactions (‘disliking’) in the same session.

### Taste Reactivity Video Scoring

Hedonic, aversive, and neutral response patterns were later scored off-line in slow motion (frame by frame to 1/10^th^ actual speed) by a trained observer who was blind to the drug condition and cannulae placement, using procedures developed to compare hedonic and aversive taste reactions [74]. Hedonic or positive responses included rhythmic midline tongue protrusions, lateral tongue protrusions, and paw licks. Aversive or negative responses included gapes, head shakes, face washes, forelimb flails, and chin rubs. Neutral responses include the relatively non-valenced behaviors or passive dripping of solution out of the mouth, ordinary grooming, and rhythmic mouth movements. All video analyses were conducted blind to drug condition and cannulae placement using Observer software (Noldus, Netherlands). A time bin scoring procedure was used to ensure that taste reactivity components of different relative frequency were balanced in their contributions to the final affective hedonic/aversive totals [74]. For example, rhythmic mouth movements, passive dripping of solution, paw licking, and grooming behaviors typically occur in long bouts, and were thus scored in 5 s time bins (up to 5 s continuous bout duration equaled one occurrence). Rhythmic tongue protrusions along the midline, which occur in shorter bouts, were scored in 2 s time bins. The other behavioral components (lateral tongue protrusions, gapes, forelimb flails, head shakes, chin rubs) typically occur as discrete events and were therefore scored as single occurrences each time they appeared (e.g., one gape scored as one occurrence). Individual totals were calculated for hedonic and aversive categories for each rat by adding all response scores within an affective category for that rat.

### Behavioral tests of fear and feeding

On a test day, rats received one of the microinjection conditions described above (DNQX, muscimol, 50% DMSO/50% 0.15 M saline, 100% 0.15 M saline) and were immediately placed in a transparent test chamber, where spontaneous behavior was videotaped for 60 min and saved for subsequent off-line analysis [4], [5]. To support eating and drinking behaviors, the chamber contained a dish of pre-weighed food chow pellets (∼20 g) and a water bottle. To support defensive treading behavior, the floor was covered with granular bedding (crushed corn cob) spread 3 cm deep. Behavior was analyzed in slow-motion by an observer blind to drug content and microinjection site in (1) eating, (2) drinking, (3) defensive treading, (4) grooming, (5) burrowing (insertion of head under corn-cob bedding, with downward and forward thrust), (6) burrow treading (combination of burrowing head thrust and paw-treading movements), (7) rearing, (8) locomotion (crossing of lines that divide six grid squares superimposed on floor of test chamber). For each behavior, a total of cumulative time (seconds) spent engaged in that action was assessed.

*Within-rat comparison*: To compare elicitation of fear versus feeding motivations on a within-subject basis to shifts in hedonic ‘liking’ versus ‘disliking’, a group of 6 rats from the taste reactivity group were selected to confirm rostrocaudal gradients of spontaneous generation of motivated eating versus fearful behaviors. After taste reactivity testing, this group was additionally tested for the elicitation of spontaneous motivated feeding behavior versus fearful treading as described above.

### Histology

After the completion of testing, rats used for behavioral testing were deeply anesthetized with sodium pentobarbital and their brains were removed and fixed in 10% paraformaldehyde overnight and then cryoprotected in 20% sucrose for at least 2 d. Brains were coronally sectioned (60 µm) mounted on slides and stained with cresyl violet or processed for Fos immunoreactivity. Cannulae placements were mapped onto drawings of the Atlas [73]. We considered a site to be rostral if it was located from +1.8 to +3.0 AP relative to bregma (including the rostral pole, between +2.4 to 3.0 mm). A site was considered to be caudal if it was located from +0.48 to +1.68 mm AP. To reveal rostrocaudal gradients more continuously, we also mapped each microinjection cannulae at its corresponding atlas location, as determined histologically, and a) continuously plotted behavior evoked at each location using color scales and b) compiled bar graphs to summarize behavioral intensities evoked at 6 rostrocaudal increments.

### Fos plume mapping procedure

Brains were processed for Fos-like immunoreactivity, 75 min after microinjections. DNQX and muscimol Fos plumes were mapped based on the percentage change in Fos-like immunoreactivity surrounding injections sites after DMQX or muscimol vs. vehicle controls, measured in blocks along each radial arm (excitatory plume  = 200% and 300% elevations above control levels; inhibitory antiplume  = 25 and 50% decline from control levels). Baselines were measured in intact brains to assess normal expression, and around the site of vehicle microinjections (Figure 2). Nearby slices were stained for Substance P to identify landmarks for comparison to a brain atlas [75].

Fos plumes were visualized by immunofluorescence processing, using NDS, goat anti-cfos and donkey anti-goat AlexaFluor 488 (excitation = 488 nm, emission = 519 nm; Molecular Probes-Invitrogen, Carlsbad, CA) as described previously [2], [3], [23], [24], [25], [76]. The size of plume symbols used for mapping was based on the average radii of Fos plumes for that drug. The color of each plume symbol was coded to show the change in behavioral effects produced by drug microinjection at the corresponding site in a particular animal. Both bilateral cannulae were plotted for each rat to depict every placement (2 sites per rat).

## Supporting Information

Figure S1 Muscimol and DNQX-induced changes in hedonic and aversive reactions. Hedonic (A) and aversive (B) reactions induced at 15 and 60 minutes post-microinjection, in saline, muscimol, DMSO/saline and DNQX drug conditions. Overall rostral rats (left) showed little overall changes in either behavior, whereas caudal rats (right) showed hedonic suppression and aversive enhancement (*p<.05; **p<.01). Error bars represent SEM.
(4.78 MB EPS)
